# The Temporal Development of Fatty Infiltrates in the Neck Muscles Following Whiplash Injury: An Association with Pain and Posttraumatic Stress

**DOI:** 10.1371/journal.pone.0021194

**Published:** 2011-06-16

**Authors:** James Elliott, Ashley Pedler, Justin Kenardy, Graham Galloway, Gwendolen Jull, Michele Sterling

**Affiliations:** 1 Division of Physiotherapy, School of Health and Rehabilitation Sciences, Centre for Clinical Research Excellence in Spinal Pain, Injury and Health, The University of Queensland, Brisbane, Australia; 2 Centre of National Research on Disability and Rehabilitation Medicine, The University of Queensland, Brisbane, Australia; 3 Centre for Advanced Imaging, The University of Queensland, Brisbane, Australia; 4 Department of Physical Therapy and Human Movement Sciences, Feinberg School of Medicine, Northwestern University, Chicago, Illinois, United States of America; University Medical Center Groningen UMCG, Netherlands

## Abstract

**Background:**

Radiological findings associated with poor recovery following whiplash injury remain elusive. Muscle fatty infiltrates (MFI) in the cervical extensors on magnetic resonance imaging (MRI) in patients with chronic pain have been observed. Their association with specific aspects of pain and psychological factors have yet to be explored longitudinally.

**Materials and Findings:**

44 subjects with whiplash injury were enrolled at 4 weeks post-injury and classified at 6 months using scores on the Neck Disability Index as recovered, mild and moderate/severe. A measure for MFI and patient self-report of pain, loss of cervical range of movement and posttraumatic stress disorder (PTSD) were collected at 4 weeks, 3 months and 6 months post-injury. The effects of time and group and the interaction of time by group on MFI were determined. We assessed the mediating effect of posttraumatic stress and cervical range of movement on the longitudinal relationship between initial pain intensity and MFI. There was no difference in MFI across all groups at enrollment. MFI values increased in the moderate/severe group and were significantly higher in comparison to the recovered and mild groups at 3 and 6 months. No differences in MFI values were found between the mild and recovered groups. Initial severity of PTSD symptoms mediated the relationship between pain intensity and MFI at 6 months. Initial ROM loss did not.

**Conclusions:**

MFI in the cervical extensors occur soon following whiplash injury and suggest the possibility for the occurrence of a more severe injury with subsequent PTSD in patients with persistent symptoms.

## Introduction

Whiplash associated disorders (WAD) are a well-documented health outcome following a motor vehicle crash (MVC). Nearly 50% report persistent symptoms up to two years post injury [1], [2]. Resultant costs for medical and rehabilitative care in the western-world are high [3]. Despite such a large socio-economic problem, reasons for the high rate of transition to chronic pain remain elusive.

Factors associated with poor recovery are not conclusive and rather limited to self-report measures. These include initial higher levels of pain, recognised as the most consistent predictor of poor outcome [4], [5], [6], with measures of sensory hyperalgesia and posttraumatic stress also showing some prognostic capacity [7], [8]. By virtue of their self-report nature, these factors are open to bias, but no verifiable structural changes (e.g. radiological findings) have shown to be associated with the transition to chronicity [9], [10]. This has contributed to the scepticism surrounding the whiplash condition [11], [12].

However, recent data has demonstrated structural muscle changes in patients with chronic WAD. Muscle fatty infiltrates (MFI) on magnetic resonance imaging (MRI) were found in the neck extensor muscles of participants with chronic WAD [13]. These findings were not present in those with chronic non-traumatic neck pain or in healthy controls [13], [14], [15]; suggesting traumatic factors play a role in their development. It is possible the presence of widespread MFI is in some way associated with the development of chronic pain following whiplash injury. As the muscle changes have been only established in chronic WAD, it is necessary to now determine how soon following injury they occur and whether they uniquely manifest in those who transition.

The underlying mechanisms contributing to the development of MFI are also unknown, but knowledge of such processes may assist in developing more informed and effective early interventions. A relationship with higher pain levels seems reasonable since the muscle changes are not apparent in non-traumatic neck pain, a condition with recognised lower levels of pain than WAD [14], [16]. The widespread findings of neck MFI suggest that disuse or lack of neck range of movement (ROM) may also be a contributing factor [17]. Additionally, stress related processes have shown to negatively affect muscle tissue [18]. Posttraumatic stress symptoms (PTSD) are common to WAD [19], [20] and associated with poor recovery [2], [21]. A significant, albeit weak, relationship between MFI and posttraumatic stress in chronic WAD has been reported [15]. The relationship between these factors at the acute stage and the development of MFI remains unknown. We therefore hypothesise that the development of MFI will be associated with initial pain levels but that this relationship may be mediated by reduced neck ROM and symptoms of PTSD.

The aims of this study were to 1) investigate the temporal development of MFI following whiplash injury, 2) investigate differences in MFI between those who recover and those who report persistent symptoms at six months post injury, 3) investigate the relationship between initial pain levels and MFI at 6 months post injury and whether this relationship is mediated by loss of neck ROM and symptoms of PTSD.

## Methods

All participants provided informed written consent. The Medical Research Ethical Committees at The University of Queensland, Australia, granted ethical approval and the study was conducted according to the principles expressed in the Declaration of Helsinki.

Fifty-five patients with acute WAD were followed and assessed from within 1 month of injury to 3 and 6 months post injury. Participants were recruited via hospital emergency departments, primary care practices and general advertisement. Participants were eligible provided they reported neck pain resulting from a motor vehicle crash (MVC) and were within the Quebec Task Force Classification category of WAD Grade II [22]. Exclusion criteria were one or more previous MVC's, treatment for neck pain disorders in the past ten years and any nervous or metabolic system disorder.

One research assistant performed the physical measures and administered questionnaires on all subjects at each assessment. The MRI measures were obtained immediately following collection of physical measures at the radiology centre by one investigator (JE) who was blind to the status of the patient in terms of questionnaire responses.

### Self-reported pain and disability

Self-reported pain and disability was measured using the Neck Disability Index (NDI) (scores reported as a percentage); which has been used to quantify pain and disability related to whiplash [23]. Subjects were also asked to rate their average pain intensity over the preceding 24 hours on a 10 cm visual analogue scale (VAS) with the anchors ‘*no pain*’ and ‘*worst pain imaginable*’.

### PTSD symptoms

Severity of PTSD symptoms was measured using total symptom severity score of the Posttraumatic Stress Diagnostic Scale (PDS) [24]. The total symptom severity score is determined across the domains of avoidance, arousal and re-experiencing the event. Higher scores indicate more severe symptoms with a maximum possible score of 51.

### Cervical range of movement

Active cervical flexion, extension and rotation (left and right) ROM was measured using a gravity goniometer (MIE Medical Research Ltd, Leeds, UK) [25]. Three recordings were obtained for each direction of movement and the mean values were used for statistical analyses. The mean values for each direction of movement were summed to give a measure of total cervical ROM (Total_ROM).

### MRI Measures and Analysis

Defined regions of interest (ROIs) were manually traced over each of the bilateral cervical extensor muscles (*rectus capitis posterior minor, major, multifidii, semispinalis cervicis, capitis, splenius capitis and upper trapezius*) on the axial T1-weighted images at each vertebral segment (C0–C7). The MFI measures were created by taking a ratio between the pixel intensities of each muscle to that of a standardized region of intermuscular fat at the C2-level [13]. Histograms were created from each muscle ROIs with MRIcro software (www.mricro.com). All axial images were acquired on 256*256 pixel matrix with a 20×20 mm field of view (TR/TE: 448/14 ms). Slice thickness was 4 mm and a dedicated flexible neck coil was used as a receiver coil. A measure for total MFI was created and used for analyses by combining and averaging the MFIs for the extensor musculature bilaterally across all cervical segments (C0–7).

### Statistical Analysis

Participants were classified based on NDI scores at 6-months post-injury as either recovered (NDI<10%), mild (NDI 10–28%) or moderate/severe (NDI≥30%). These classifications have been used previously [26] and supported in subsequent works [2].

A repeated measures linear mixed model was used to determine the effects of time and group and the interaction effect of time * group on MFI. Age and gender were included as covariates to account for their potential to influence the relationship between pain and MFI [15]. Planned comparisons were performed to assess differences within and between groups. Sidak adjustments were used to control for Type I error rate. P<0.05 was considered statistically significant. All tests were 2-tailed. Data analyses were performed using SPSS v. 18 (Chicago, Illinois, USA). Based on our earlier data of groups differences in MFI [13], and a between group effect size of 1.2, 12 subjects/group were required at 80% power and p = 0.05.

**Figure 1:**
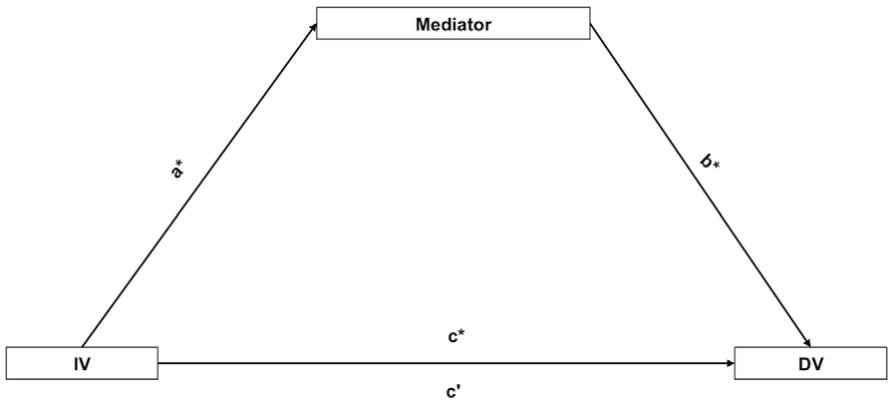
Relationship between the independent variable (IV), dependent variable (DV) and mediator variable. **a** = effect of IV on mediator, **b** = effect of mediator on DV after controlling for IV, **c** = effect of IV on DV and **c'** = effect of IV on DV after controlling for the mediator. Effects indicated by * must be significant and **c'** must be non-significant for mediation hypothesis to be confirmed.

**Figure 2:**
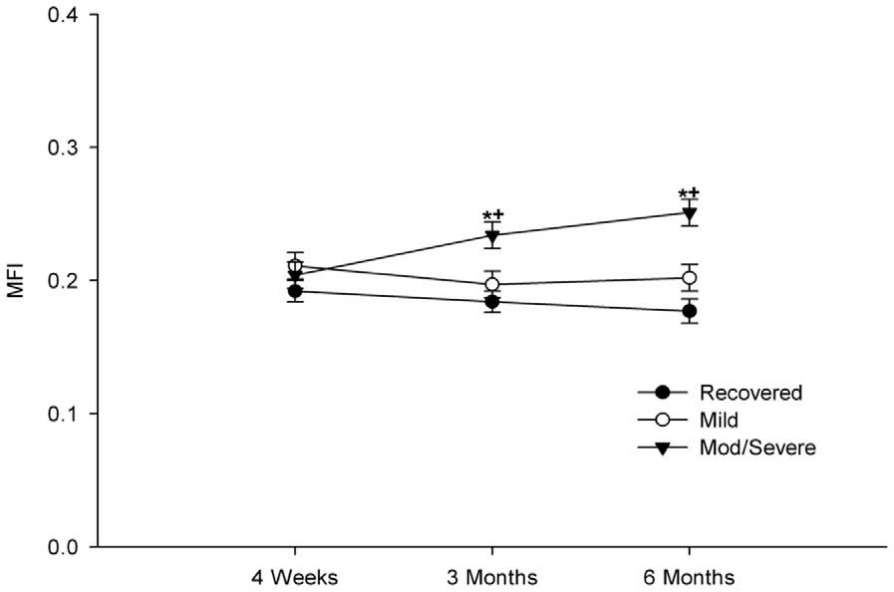
Changes in MFI over time in each group. * indicates significantly different from the recovered group at p<0.05 level. **+** indicates significantly different from the mild group at the p<0.05 level.

We conducted separate analyses to determine whether the relationship between baseline pain intensity and MFI at 6-months was mediated by initial baseline measures of PTSD symptoms and Total_ROM. In order for a variable to be classified as exerting a meditational effect on the relationship between an independent (initial pain intensity) and dependent variable (MFI at 6-months) the following criteria must be met: 1) the independent variable must significantly predict the proposed mediator variable (path **a** in Figure 1), 2) the independent variable must significantly predict the dependent variable (path **c** in Figure 1), 3) the proposed mediator must significantly predict the dependent variable controlling for the effect of the independent variable (path **b** in Figure 1) and 4) the influence of the independent variable on the dependent variable must be reduced after controlling for the mediator (path **c'** in Figure 1) [27]. The mediation effect is stronger when path **c'** approaches zero.

In each mediation analysis the unstandardised regression coefficients for each of the relationships described in Figure 1 were calculated and tested for significance. In order to test if the indirect effect of the independent variable (acting through the mediator variable) on the dependent variable was non-zero, 95% confidence intervals with a bias-corrected, accelerated bootstrapping technique was used [28]. Confidence intervals were created through computation of 5000 estimates of the indirect effect and determination of the 2.5% highest and lowest scores of the empirical distribution of these estimates. The indirect (mediational) effect was considered significant when the bias-corrected confidence intervals did not include zero.

## Results

Participant demographics are shown in Table 1. Of the 55 enrolled participants, 2 (4%), per institutional safety MRI guidelines, were excluded from further analysis. A further 6 subjects (10%) withdrew from the study. During the course of the study, 2 subjects (4%) relocated and 1 (2%) fell pregnant and were thus unable to complete all time points. Accordingly, 44 subjects (80%) completed all of the MRI measures.

**Table 1:** Demographics of subject groups at 6 months according to the Neck Disability Index (Vernon and Mior, 1996).

Group	N	Age (years)(mean ± SD)	Gender (% female)	BMI(mean ± SD)	NDI Classification	NDI (mean ± SD)	PDS(mean ± SD)	Total_ROM °(mean ± SD)
**Recovered**	17	28.8 (7.4)	77.8	25.1 (4.5)	<8%	3.1 (3.6)	4.9 (7.5)	181.4 (34.0)
**Mild**	15	36.7 (9.7)	68.8	28.5 (5.6)	10–28%	17.4 (6.8)	10.4 (8.7)	158.2 (45.9)
**Moderate/Severe**	12	37.3 (9.2)	76.9	27.8 (6.1)	>30	44.1 (7.1)	17.6 (12.3)	136.2 (30.8)

### MRI Findings

Repeated measures analysis showed a significant main effect for group (F [2, 40.7] = 7,9, p = 0.001) and a significant interaction effect of group*time (F [4, 65.7] = 4.3, p = 0.001) on MFI. Gender was also found to have a significant effect on MFI (F [1, 39.4] = 4.9, p = 0.033). Planned comparisons showed no significant differences in MFI between groups at baseline recovered vs. mild p = 0.368, recovered vs. mod/severe p = 0.763, mild vs. mod/severe p = 0.921. Between baseline and 3 months post-injury, the moderate/severe group uniquely displayed a significant increase in MFI (Table 2) (p = 0.002), and at this point had significantly higher MFI than the recovered (p = 0.001) and mild groups (p = 0.021). These differences persisted at 6 months follow up (Figure 2).

**Table 2:** Estimated marginal means of MFI data for all three groups over time (mean [95% CI])* indicates significant within group differences between 4 weeks and 3 months.

	4 weeks	3 months	6 months
TOTAL_MFI			
**Recovered**	0.192[0.176, 0.208]	0.184[0.167, 0.200]	0.177[0.159, 0.194]
**Mild**	0.211[0.192, 0.230]	0.197[0.177, 0.216]	0.202[0.181, 0.222]
**Moderate/Severe**	0.204[0.184, 0.223]	0.234*[0.215, 0.254]	0.251*[0.231, 0.270]

### Mediation Analyses

**Table 3** details the results of the mediation analyses. Having higher baseline pain was associated with having increased MFI at 6 months post-injury (**c** in **Table 3**). In addition, higher baseline pain intensity was also associated with having PTSD symptoms and reduced cervical spine ROM (**b** in **Table 3**). When the effect of the mediator variable was controlled for, the effect of baseline pain intensity on MFI at 6 months was reduced and no longer significant in both models (**c'** in **Table 3**). However, when the effect of baseline pain intensity was controlled for, PTSD symptoms had a significant positive association with MFI at 6 months while Total_ROM did not (**b** in **Table 3**). Point estimates and 95% confidence intervals of the mediation effect confirmed that the effect of baseline pain intensity on MFI acting through PTSD was significantly different from 0 allowing acceptance of the hypothesis that PTSD is a partial mediator of the relationship between baseline pain intensity and MFI at 6-months.

**Table 3:** Unstandardised regression coefficients for pathways in mediation analyses where regression coefficients a, b, c, c' reflect the pathways described in Figure 1.

Model	Mediator	Regression Coefficients				MediationEffect	Bootstrapping 95% CI	
		A	b	c	c'	Estimate	Lower	Upper
**1**	PTSD	1.86*	0.002*	0.006*	0.003	0.003	0.001	0.007
**2**	Total_ROM	−6.11*	−0.003	0.006*	0.004	0.002	−0.001	0.007

The independent variable is baseline pain intensity and the dependent variable is MFI at 6 months follow up for each model.

*Denotes coefficients which are statistically significant at the p>0.05 level. Mediation effect represents the point estimate of the indirect effect of the independent variable on the dependent variable acting through the mediator calculated through bias-corrected accelerated bootstrapping. Mediation effect is significant where the 95% CI do not span 0.

## Discussion

Our study provides the first objective evidence for the differential development of MFI in participants with varying levels of functional recovery following whiplash injury. Previous investigations of whiplash have attempted to identify and link structural pathology with symptoms but without consistent results [9], [10], [29], [30], [31]. However, these studies did not focus on muscular tissues. This study has uniquely 1) identified longitudinal structural muscle pathology with MRI between 4-weeks and 3-months post injury, 2) used these findings to differentiate between those with varying levels of functional recovery, and 3) shown that the relationship between MFI at 6-months post injury and initial pain intensity is mediated by PTSD symptoms.

All of the groups entered the study at 4-week post-injury with similar levels of MFI. However, the group with poor functional recovery at 6-months uniquely demonstrated increased MFI between 4-weeks and 3-months post-injury and these changes persisted at 6-months. The participants reporting mild pain and disability and recovery did not demonstrate such changes. This is interesting and suggests the possibility of a more severe injury in those with poor functional recovery. There are various processes that could underlie the development of such muscle degeneration and these require consideration.

Physiologic response to injured cervical structures includes profound neuroinflammatory changes in the dorsal root ganglia and spinal cord [32], [33], which, similar to other chronic pain disorders [34], could lead to persistent pain states in WAD. While this study did not explore the specific role of inflammation on outcomes, the widespread muscle changes and associated higher pain could be consistent with a local and/or systemic inflammatory response from damaged somatic structures and attendant alterations in pain processing from and within the peripheral and/or central nervous systems [35].

It remains possible that the expression of fat cells is the result of an injury induced inflammatory response and the subsequent increase in DNA synthesis of the many different cells within the peri-muscular connective tissue e.g. mast cells, satellite cells, muscle precursor cells, fibroblasts and preadipocytes. These cells, after injury, are responsible for secreting pro-inflammatory cytokines that could stimulate their trans-differentiation into adipose tissue [36], [37], [38], [39]. While causal inference between MFI and poor recovery cannot be drawn from the results of this study, it is possible that the presence of MFI (which is not patient dependent), is the result of an initial and persistent inflammatory response [40] to injured anatomical tissues. As such, changes in muscle structure may represent an objective MRI marker for injury. Further study investigating the influence of an early and persistent local and/or systemic inflammatory response to whiplash injury on neck MFI development and the transition to chronic pain is warranted and well underway.

Complex, physiological factors related to disuse may also stimulate and produce structural muscle changes [17], [41], [42]. Postural muscles demonstrate rapid accumulations of intermuscular fat after 4-weeks of disuse [17]. Reduced cervical ROM following whiplash is common and associated with higher levels of pain and poor functional recovery [2], [26]. We therefore tested a potential disuse model where reduced cervical ROM mediated the effect of initial pain intensity on the development of MFI. While initial pain intensity significantly influenced the development of MFI at 6-months, the mediational effect of reduced ROM in this relationship was not supported. Suggesting that pain and ROM interact separately and/or possibly in tandem through other biopsychosocial pathways [43].

On the contrary, the relationship between high initial pain and MFI was mediated by the presence of PTSD symptoms at 4-weeks post-injury. To our knowledge, this is the first study on whiplash to demonstrate a relationship between symptoms of PTSD (a psychological finding) and objective longitudinal data for muscle degeneration (a physical pathology). Support for a psychobiologic link is available. Induced sympathetic nervous system activation that may occur in tandem with posttraumatic stress symptoms is centrally programmed and has shown to exert a number of actions at the muscle level [18]. While these actions are beneficial in the acute stage of stress, prolonged stress can negatively impact the muscle system [18]. Excessive sympathetic outflow can produce vasoconstriction resulting in metabolic consequences such as hypoxia and toxaemia. Under this condition, intra-myocellular oxidative stresses may be responsible for inducing muscle damage [44]. The persistent presence of oxidative stress can dramatically affect the contractility of skeletal muscle as well as induce fibrotic degeneration, commonly seen in other painful conditions (e.g. fibromyalgia) and possibly the fatty muscle changes observed in this study.

Furthermore, this may be especially important for some cases of traumatic whiplash when considering the potential for even greater consequences on health outcomes as has been observed in other populations with PTSD [21], [45], [46]. For example, exposure to extreme stress can activate various biological processes including the release of cortisol by the adrenal glands. Sustained hypercortisolemia can profoundly impact overall health status, including the degeneration of skeletal muscle [42]. Additional biological consequences associated with exposure to extreme stress include autonomic reactivity, disturbed sleep, lowered immunity and altered perception of symptoms [47]. A potential conclusion to be drawn on the basis of such information is that increased levels of MFI could be related to alterations of the neuroendocrine system in some patients following whiplash, suggesting a neuro-psycho-biological link with poor outcomes. Further research to investigate the activation of various biological processes in tandem with stress system responses following whiplash is warranted and required.

This is the first study to demonstrate an association between structural muscle changes and transition to chronicity in WAD. These changes were predicted by higher initial pain intensity and the effect of pain intensity on MFI was mediated by symptoms of PTSD. While this study utilized a relatively small sample, the low variability found within groups (Figure 2, Table 2) supports the strength of the MRI measure for detecting group differences in MFI. The results of this study can therefore be interpreted with confidence in the statistical power of our analyses.

These findings may therefore have implications for the whiplash condition where the early assessment and management of stress responses may attenuate some physical aspects of the condition. This and other questions related to the origin of muscle degeneration following traumatic whiplash injury and the overall contribution to long-term outcomes requires further evaluation.
